# Transactivated Epidermal Growth Factor Receptor Recruitment of α-actinin-4 From F-actin Contributes to Invasion of Brain Microvascular Endothelial Cells by Meningitic *Escherichia coli*

**DOI:** 10.3389/fcimb.2018.00448

**Published:** 2019-01-09

**Authors:** Jiyang Fu, Liang Li, Xiaopei Yang, Ruicheng Yang, Nouman Amjad, Lu Liu, Chen Tan, Huanchun Chen, Xiangru Wang

**Affiliations:** ^1^State Key Laboratory of Agricultural Microbiology, College of Veterinary Medicine, Huazhong Agricultural University, Wuhan, China; ^2^Key Laboratory of Preventive Veterinary Medicine in Hubei Province, The Cooperative Innovation Center for Sustainable Pig Production, Wuhan, China; ^3^Key Laboratory of Development of Veterinary Diagnostic Products, Ministry of Agriculture of the People's Republic of China, Wuhan, China; ^4^International Research Center for Animal Disease, Ministry of Science and Technology of the People's Republic of China, Wuhan, China

**Keywords:** bacterial meningitis, epidermal growth factor receptor, α-actinin-4, cytoskeleton, invasion

## Abstract

Bacterial penetration of the blood-brain barrier requires its successful invasion of brain microvascular endothelial cells (BMECs), and host actin cytoskeleton rearrangement in these cells is a key prerequisite for this process. We have reported previously that meningitic *Escherichia coli* can induce the activation of host's epidermal growth factor receptor (EGFR) to facilitate its invasion of BMECs. However, it is unknown how EGFR specifically functions during this invasion process. Here, we identified an important EGFR-interacting protein, α-actinin-4 (ACTN4), which is involved in maintaining and regulating the actin cytoskeleton. We observed that transactivated-EGFR competitively recruited ACTN4 from intracellular F-actin fibers to disrupt the cytoskeleton, thus facilitating bacterial invasion of BMECs. Strikingly, this mechanism operated not only for meningitic *E. coli*, but also for infections with *Streptococcus suis*, a Gram-positive meningitis-causing bacterial pathogen, thus revealing a common mechanism hijacked by these meningitic pathogens where EGFR competitively recruits ACTN4. Ever rising levels of antibiotic-resistant bacteria and the emergence of their extended-spectrum antimicrobial-resistant counterparts remind us that EGFR could act as an alternative non-antibiotic target to better prevent and control bacterial meningitis.

## Introduction

Bacterial meningitis is the most important life-threatening infection to attack the central nervous system (CNS), and is associated with high morbidity and mortality (Kim, 2010; Koedel et al., 2010; Agrawal and Nadel, 2011). In some developing countries and regions with poor medical conditions, the incidence of bacterial meningitis can reach 1,000 cases per 100,000 people per year, with mortality rates reaching up to 30% even with advanced sanitation and antimicrobial treatment (McGill et al., 2016). Its irreversible destruction of the CNS means that ~50% of the survivors (infant survivors especially) suffer from multiple neurological sequelae (Kim, 2003). Consequently, bacterial meningitis is now recognized as an important cause of infection-associated deaths worldwide (Kim, 2010; McGill et al., 2016).

Many bacterial pathogens are able to invade the CNS and cause damage to the host, among which *Escherichia coli* is the most common Gram-negative bacillary organism causing meningitis, especially in infants and young children (Kim, 2008, 2016; Zelmer et al., 2008; Wang et al., 2016; Yang et al., 2016a,b; Coureuil et al., 2017). Most cases of *E. coli* meningitis are initiated *via* hematogenous spread and develop from bacterial penetration of the blood–brain barrier (BBB), which is an essential prerequisite for *E. coli*-induced CNS infection (Kim, 2003; Wang et al., 2016). Three main routes have been proposed for how bacterial pathogens traverse the BBB: transcellular, paracellular, and the so-called “Trojan horse” mechanism through which bacterial pathogens hide in infected phagocytic cells (Kim, 2008; Coureuil et al., 2017). Evidence from *in vivo* and *in vitro* studies supports the notion that meningitic *E. coli* (mainly the *E. coli* K1-capsule strain) traverses the BBB mainly through a transcellular mechanism whereby the actin cytoskeleton in the brain microvascular endothelial cells (BMECs) becomes rearranged (Kim, 2002, 2008; Sukumaran et al., 2002; Rudrabhatla et al., 2006). However, as far as we are aware, the specific molecules involved in cytoskeleton rearrangement in BMECs have not been fully identified, and the detailed intracellular events involved in this process in response to meningitic *E. coli* infection are yet to be elucidated.

Epidermal growth factor receptor (EGFR) belongs to the family of receptor tyrosine kinases and is known to be an important regulator of multiple cellular processes in cancer-related diseases, as well as in pain processing and chronic kidney disease (Wang and Kim, 2002; Linggi and Carpenter, 2006; Chen et al., 2017; Martin et al., 2017). To date, several bacterial pathogens have been reported to target EGFR through different mechanisms to facilitate their infection of host cells, including *Neisseria gonorrhoeae, N. meningitidis, Helicobacter pylori, Haemophilus influenzae*, and *Klebsiella pneumonia* (Mikami et al., 2005; Yan et al., 2009; Swanson et al., 2011; Edwards et al., 2013; Frank et al., 2013). In our previous work on chemical library screening and *in vitro* human BMECs (hBMECs) invasion assays, we showed that the meningitic *E. coli* K1-capsule strain could exploit EGFR activation for its invasion of the BBB (Wang et al., 2016). However, whether activated EGFR can regulate the actin cytoskeleton in BMECs and thus facilitate bacterial invasion is still unclear. Therefore, to better address this issue, the key molecules that are involved in maintaining and regulating the actin cytoskeleton should also be identified.

α-actinin (ACTN), a member of the spectrin family of cytoskeletal proteins, was first recognized as an actin cross-linking protein (Li et al., 2016). This protein has been implicated in a broad range of cytoskeleton-associated cellular processes such as those involving cellular adhesion, migration, immune cell targeting, and drug-resistance, because it forms an antiparallel homodimer with an actin binding head at the amino terminus of each monomer (Otey and Carpen, 2004). Four ACTN isoforms have been reported, among which the muscle isoforms ACTN2 and ACTN3 are localized mainly in skeletal, cardiac, and smooth muscle fibers, while the cytoskeletal isoforms ACTN1 and ACTN4 are ubiquitously present in multiple cellular structures such as stress fibers, adhesion or intercellular contact sites, filopodia and lamellipodia, for example (Hsu and Kao, 2013). Previous work with HeLa cells showed that the Na^+^/H^+^ exchanger regulatory factor played a role in actin cytoskeleton disassembly by increasing ACTN4 ubiquitination and decreasing its expression levels (Sun et al., 2016). Moreover, the interaction of ACTNs with intercellular adhesion molecule-5 (ICAM-5) helps with cytoskeleton anchorage and promotes neurite outgrowth (Nyman-Huttunen et al., 2006). In osteoblasts, the interaction of ACTNs with integrins stabilizes the focal adhesions and protects cells from apoptosis (Triplett and Pavalko, 2006). Currently, the question as to whether ACTNs participate in altering the host actin cytoskeleton, thereby contributing to meningitic *E. coli* invasion of the BBB remains open, as is the underlying mechanism concerning ACTNs in this process.

Thus, in the present study, we sought to investigate the specific role of EGFR, and the potential interrelationship between EGFR and cytoskeleton-associated protein ACTNs during meningitic *E. coli* invasion of the BBB. We have already shown that an *E. coli* strain with a K2-capsule was able to penetrate the BBB and cause meningitis, just like a K1-capsule strain (Liu et al., 2015; Yang et al., 2016b). Therefore, we used a K2-capsular *E. coli* strain (PCN033) isolated from the cerebrospinal fluid of a case of porcine meningitis (Liu et al., 2015), to explore the potential function as well as the detailed mechanism of the EGFR-ACTN interaction in bacterial penetration of the BBB. Our results support the key role of EGFR as well as a competitive recruitment mechanism involving EGFR-ACTN4 interaction in meningitic *E. coli* invasion of the BBB. Notably, this mechanism is not only shared by both meningitic *E. coli* K1-capsular and K2-capsular strains, but is also commonly hijacked by *Streptococcus suis*, a Gram-positive bacterial pathogen with the ability to cause meningitis in humans and other animals. Transactivated EGFR recruitment of ACTN4, therefore, is likely to be a common mechanism utilized by multiple CNS-infecting bacterial pathogens for BBB penetration, and these molecules are potential targets for future prevention and therapy of bacterial meningitis.

## Materials and Methods

### Bacterial Strains and Cell Culture

*E. coli* K2-capsular strain PCN033 is a swine cerebrospinal fluid isolate (Liu et al., 2015). *E. coli* K1-capsular strain RS218 (O18:K1:H7) was originally obtained from the cerebrospinal fluid of a neonate with meningitis (Huang et al., 1995), and gifted from Prof. Kwang Sik Kim in Johns Hopkins University School of Medicine. *E. coli* K12 strain HB101 was used as the meningitis-negative strain (Khan et al., 2007). *S. suis* serotype 2 strain SC19 was isolated from a swine brain during the *S. suis* outbreak in Sichuan Province in China in 2005 (Zhang et al., 2016). The *E. coli* strains were grown aerobically at 37°C in Luria–Bertani medium unless otherwise specified. SC19 was cultured in tryptic soy broth (Difco Laboratories, Detroit, MI, USA) medium with 10% newborn bovine serum at 37°C unless otherwise specified. Bacterial strains were incubated in a water bath at 65°C for 30 min for heat inactivation.

The hBMECs were gifted from Prof. Kwang Sik Kim in Johns Hopkins University School of Medicine, and routinely cultured in RPMI 1640 supplemented with 10% fetal bovine serum, 2 mM L-glutamine, 1 mM sodium pyruvate, essential amino acids, nonessential amino acids, vitamins, and penicillin and streptomycin (100 U/mL) in a 37°C incubator under 5% CO_2_ until monolayer confluence was reached. In some experiments, confluent hBMECs were washed thrice with Hank's Balanced Salt Solution (Corning Cellgro, Manassas, VA, USA) and starved in serum-free medium (1:1 mixture of Ham's F-12 and M-199) for 12–16 h before further treatment. As specified in some assays, the cells were pretreated with various inhibitors prior to adding the bacteria.

### Reagents, Antibodies and Plasmids

The EGFR tyrosine kinase inhibitor AG1478 and Gefitinib, the ErbB2 inhibitor AG825, the ADAM17 selective inhibitor TAPI-1 were purchased from Medchem express (Princeton, NJ, USA), and the HB-EGF inhibitor CRM197 was purchased from Pfenex Inc. (San Diego, CA, USA). The immunofluorescence staining kit containing Cy3-labeled goat anti-rabbit IgG, the immunofluorescence staining kit containing Cy3-labeled donkey anti-goat IgG, the immunofluorescence staining kit containing FITC-labeled goat anti-rabbit IgG, the immunofluorescence staining kit containing DyLight 405-labeled goat anti-mouse IgG, actin-tracker green conjugated with FITC, protein A + G agarose beads and CCK-8 assay kit were all obtained from Beyotime (Shanghai, China). The horseradish peroxidase (HRP)-conjugated anti-phosphotyrosine (4G10) antibody was purchased from Merck Millipore Corporation (Temecula, CA, USA). Anti-EGFR antibody was purchased from Antibody Revolution Inc. (San Diego, CA, USA). Anti-ErbB2 and anti-ErbB3 antibodies, HRP-conjugated anti-rabbit IgG antibody, and HRP-conjugated anti-mouse IgG antibody were all purchased from Cell Signaling Technology (Danvers, MA, USA). Anti-ErbB4 and anti-ACTN4 antibodies were purchased from Proteintech (Chicago, IL, USA). Anti-*E. coli* antibody was purchased from Abcam (Cambridge, MA, USA). Anti-β-actin antibody was obtained from Hua An Biotechnology Co., Ltd. (Hangzhou, China). The lipofectamine 3000 transfection reagent was obtained from Invitrogen (Carlsbad, CA, USA). Puromycin was purchased from Corning (Corning, NY, USA). The ErbB3 shRNA plasmid and the control shRNA plasmid-A were purchased from Santa Cruz Biotechnology (Dallas, TX, USA). EGFR and ACTN4 CRISPR/Cas9 plasmids were purchased from Nanjing YSY Biotech Co. LTD. (Nanjing, China).

### Bacterial Invasion of hBMECs

The *in vitro* ability of *E. coli* strains to invade hBMECs was determined according to previous methodologies (Wang et al., 2016; Yang et al., 2016b). Briefly, PCN033 or RS218 was cultured overnight, and resuspended in the experimental medium (M199-Ham F12 [1:1] medium containing 5% FBS). The confluent hBMECs growing in 24-well plates were challenged with bacteria at a multiplicity of infection (MOI) of 100 (~5 × 10^7^ CFU/well) to allow invasion at 37°C for 90 min. The cells were then washed thrice to remove free bacteria and incubated in medium containing specific antibiotics for 2 h to kill the extracellular bacteria. Finally, the cells were extensively washed and lysed with 0.025% Triton X-100 buffer to release the intracellular bacteria. The bacteria were quantified by serial dilutions and plating. The results were calculated as percentages of the initial inoculums and presented here as the percentage relative invasion compared with that of the control group. Each invasion assay was performed three times and results of each time were obtained with three duplications.

### CCK-8 Assay

The CCK-8 assay was performed according to the manufactures' instructions (Beyotime, Shanghai, China). The hBMECs were seeded in 96-well plate at 5 × 10^3^ per well and incubated overnight. The chemicals and the CCK-8 reagent were added in the cells and incubated at 37°C for indicated time. The absorbance of each well was measured at 450 nm. Each treatment was performed in triplicate. Results were recorded as mean ± standard deviation (mean ± SD).

### Immunoprecipitation and Western Blotting

The hBMECs were seeded at 1 × 10^6^ cells in 100 mm dishes. The confluent hBMECs were serum-starved and then challenged with bacteria at a MOI of 10 for the times indicated, and the cell lysates were then collected for immunoprecipitation and western blotting analyses as previously described (Hoffmann et al., 2001). The blots were visualized with ECL reagents, and densitometric analysis was performed using Image Lab software (Bio-Rad, Hercules, CA, USA).

### RNA Isolation and Quantitative Real-Time PCR Analysis

Total cellular RNA was extracted using TRIzol reagent (Invitrogen), and cDNA was prepared using the PrimeScript^TM^ RT reagent kit with gDNA Eraser (Takara Bio Inc., Shiga, Japan). Real-time PCR was performed with a qTOWER^3^/G quantitative real-time PCR thermal cycler (Analytikjena, Jena, Germany) using Power SYBR Green PCR master mix (Applied Biosystems, Foster City, CA, USA) according to the manufacturer's instructions. The primers used for the real-time PCRs were listed in Supplementary Table 1. The amplification conditions were 50°C for 2 min, 95°C for 10 min, followed by 40 cycles of 95°C for 15 s and 60°C for 1 min. The products were subjected to a melting curve stage comprising denaturation at 95°C for 15 s, annealing at 60°C for 1 min, and slow dissociation by ramping from 60 to 95°C at 0.1°C/s to ensure the specificity of the primers for their target sequences. Expression of the target genes was normalized against *GAPDH*. Each assay was performed in triplicate.

### Immunofluorescence

Cells were seeded and grown in glass-bottomed dishes (Ø 35 mm) for 12 h, and then challenged with bacteria at a MOI of 10 for 3 h. Immunofluorescence experiments were performed according to the instructions provided by the relevant staining kits. Briefly, the cells were washed with PBS three times and then fixed with 4% paraformaldehyde for 30 min. The fixed cells were then treated with 1% Triton X-100 in PBS prior to non-specific site blocking and antibody incubation. Here, ACTN4 was labeled with Dylight 405, EGFR was labeled with Cy3, and F-actin was stained with actin-tracker green. The cells in the dishes were finally mounted and visualized with a Zeiss LSM 880 microscope (Carl Zeiss Jena, Jena, Germany). The gray scale images exporting and the cellular fluorescence density analysis were performed with ImageJ software.

### Secretory HB-EGF Determination by ELISA

Cells were seeded at 1 × 10^5^ in 24-well plates and then cultured until confluence. The cells were then serum-starved and stimulated with meningitic *E. coli* PCN033 at a MOI of 10 for 3 h, and the supernatant was collected *via* centrifugation to obtain the free HB-EGF. Also, the HB-EGF attached to the cells was dissolved with washing buffer and then collected as described previously (Wang et al., 2016). The secretory HB-EGF in the cells in response to the bacterial challenge was quantified using the HB-EGF ELISA Kit (Abcam, Cambridge, MA, USA) following the procedures indicated by the manufacturer.

### Transfection

hBMECs grown to 70% confluence were used with the Lipofectamine 3000 reagent (Invitrogen) for the transfection experiments following the manufacturer's instructions. Briefly, 5 μg of plasmid, 10 μL of P3000, 7.5 μL of Lipo3000 and 500 μL of Opti-MEM were mixed gently and then incubated at room temperature for 15 min. The mixture was then added dropwise to the cells in the 6-well plates followed by incubation at 37°C with 5% CO_2_ for 24 h. Fresh medium containing puromycin (100 μg/ml) was then used to screen the cells and maintain those that were transfection-positive.

### CRISPR/Cas9 Genomic Editing

The hBMECs were transfected with commercially synthesized CRISPR/Cas9 plasmids containing puromycin resistance gene as mentioned above. After 24 h of transfection, the medium was replaced and positive-transfected cells were selected in the fresh medium containing puromycin for another 24 h. Surviving cells were transferred into 96-well plates with limiting dilution and incubated until single-cell clone was formed. The genomic DNA from each cell clone was extracted and subjected to PCR amplification, and positive editing cells were identified through the sequencing.

### Statistical Analysis

Data are expressed as the mean ± standard deviation (mean ± SD). Statistical significance of the differences between each group was analyzed by a one-way analysis of variance (ANOVA) or two-way ANOVA embedded in GraphPad Prism, version 6.0 (GraphPad Software Inc., La Jolla, CA, USA). Differences between the *in vitro* bacterial invasion rates were determined by Student's *t*-test. *P* < 0.05 (^*^) was considered significant, and *p* < 0.01 (^**^) was considered extremely significant.

## Results

### Activation of EGFR in hBMECs Induced by Meningitic *E. coli* K2 Largely Contributes to Its Invasion

**Figure 1:**
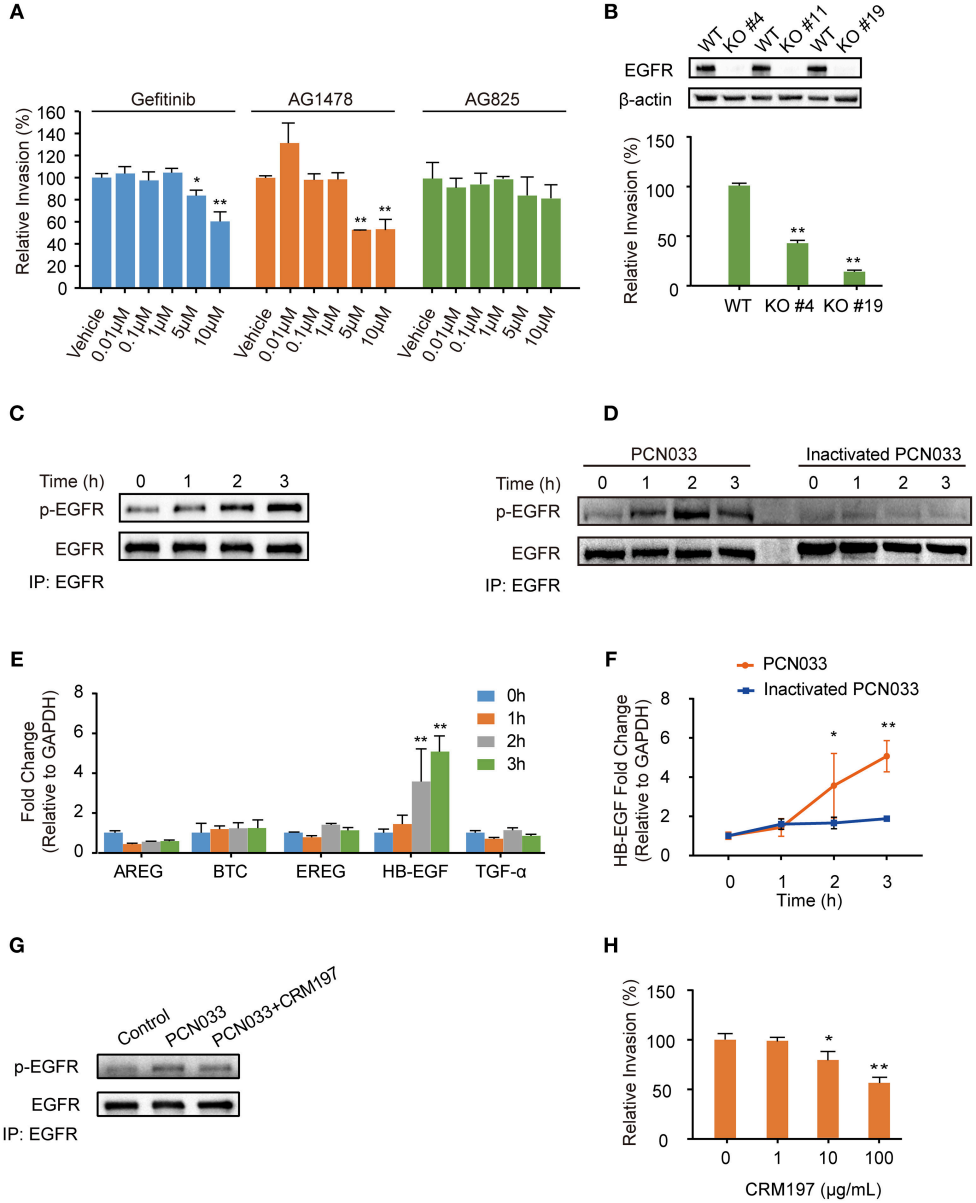
Meningitic *E. coli* PCN033-induced activation of EGFR contributes to its invasion of hBMECs. **(A)** Effects of the EGFR inhibitor gefitinib, AG1478 and the analog AG825 on PCN033 invasion of hBMECs. Data represent the mean ± SD of three duplications and are presented here as relative invasion compared with the vehicle control. **(B)** Verification of successful EGFR KO and its influence on bacterial invasion. **(C)** Tyrosine phosphorylation of EGFR during the infection. **(D)** EGFR activation in response to both viable PCN033 and heat-inactivated PCN033 strains. **(E)** Quantitation analysis of different EGFR ligands in response to PCN033 infection. GAPDH was used as the endogenous control. **(F)** Comparison of the induction of HB-EGF by the viable and inactivated PCN033 over time. **(G)** Effect of CRM197 treatment on the PCN033-induced EGFR activation. hBMECs were challenged with PCN033 for 3 h. **(H)** The effect of CRM197 on PCN033 invasion of hBMECs. The results represent the relative invasion percentage compared with the untreated control. ^*^p < 0.05, ^**^p < 0.01.

Current scientific understanding largely supports that K1-capsular *E. coli* can invade BMECs and traverse the BBB effectively, and our previous work has also reported that the meningitic *E. coli* K1 strain can hijack EGFR activation to facilitate its invasion of hBMECs (Wang et al., 2016). Notably, we have published a large amount of evidence showing that K2-capsular *E. coli* strains can invade hBMECs and cause disruption of the BBB, thereby leading to meningitis (Yang et al., 2016b). But there is an open question about whether the K2-capsular strains can also exploit EGFR activation, such as that observed in the K1-capsular strain, for successful cell invasion. To address this question, we firstly used two selective EGFR inhibitors, gefitinib and AG1478, as well as AG825 (an analog of AG1478 that selectively inhibits the activation of ErbB2) to investigate the possible role for EGFR in *E. coli* K2 (PCN033 strain) invasion of hBMECs. We observed a significant decrease in bacterial invasion with gefitinib or AG1478 treatment in a dose-dependent manner, but AG825 treatment did not decrease bacterial invasion (Figure 1A). As a supplement, we demonstrated that gefitinib, AG1478 and AG825 at indicated concentrations did not affect bacterial growth (Supplementary Figures 1A–C), nor have toxic effect on hBMECs (Supplementary Figures 2A–C). Next, we generated EGFR knock-out (KO) cell lines, and compared bacterial invasion of the wild-type and EGFR-KO cells. The results showed that invasion of the EGFR-KO cells by the K2-capsular PCN033 strain decreased significantly when compared with that of the wild-type cells (Figure 1B). This result indicates a potential role for EGFR in facilitating *E. coli* K2 invasion. We next observed that EGFR could be tyrosine-phosphorylated in response to PCN033 challenge over time (within 3 h, Figure 1C), and the inactivated strain was unable to induce EGFR phosphorylation (Figure 1D). Moreover, we found that when viable, the K2 PCN033 strain could induce a time-dependent increase in transcription of the EGFR-ligand, HB-EGF, while the inactivated bacteria could not (Figures 1E,F). When HB-EGF was blocked by CRM197, a nontoxic mutant of the diphtheria toxin that binds to proHB-EGF and prevents its shedding from EGFR stimulation (Thornton et al., 2015; Nam et al., 2016), the PCN033-induced activation of EGFR was significantly inhibited (Figure 1G), while PCN033 invasion of hBMECs showed a concomitant decrease following CRM197 treatment in a dose-dependent manner (Figure 1H). The CRM197 did not show any toxicity on the growth of *E. coli* (Supplementary Figure 1D) or hBMECs (Supplementary Figure 2D). All these observations are highly consistent with our previous findings in the meningitic *E. coli* K1 strain (Wang et al., 2016), raising the concept that meningitic *E. coli* strains with K1 or K2 capsules can exploit the same host target, EGFR, for their invasion of hBMECs.

### ADAM17-Mediated HB-EGF Release in hBMECs Is Responsible for Meningitic *E. coli* Activation of EGFR

HB-EGF is synthesized initially as a membrane-spanning precursor molecule and proteolytically processed by metalloproteinases of the ADAM (a disintegrin and metalloproteinase) family (Zhuang et al., 2008; Martín et al., 2012; Taylor et al., 2014). Our previous whole transcription data indicated that three ADAM family members, ADAM15, ADAM17 and ADAM19, were expressed in hBMECs (Yang et al., 2016a). Hence, we next checked for expressional changes in them in response to meningitic *E. coli* challenge. We found that the levels of ADAM17, not ADAM15 or ADAM19, increased significantly upon infection (Supplementary Figure 3A). To further verify the involvement of ADAM17, we treated the cells with TAPI-1, a specific inhibitor of ADAM17 (Raikwar et al., 2014), prior to PCN033 infection, and found that the time-dependent phosphorylation of EGFR induced by PCN033 was completely blocked by TAPI-1 (Supplementary Figure 3B). We also observed that blocking ADAM17 by TAPI-1 did not affect the PCN033-induced transcriptional upregulation of HB-EGF, while the secretion of HB-EGF protein was prevented markedly (Supplementary Figures 3C,D). We did not observe the toxic effects of TAPI-1 on both bacteria (Supplementary Figure 1E) and the cells (Supplementary Figure 2E). These findings support the involvement of ADAM17 in meningitic *E. coli*-induced EGFR activation in hBMECs, *via* its proteolytic cleavage of HB-EGF.

### *E. coli*-Induced Dimerization of EGFR-ErbB3 Is Important for EGFR Activation and Bacterial Invasion

**Figure 2:**
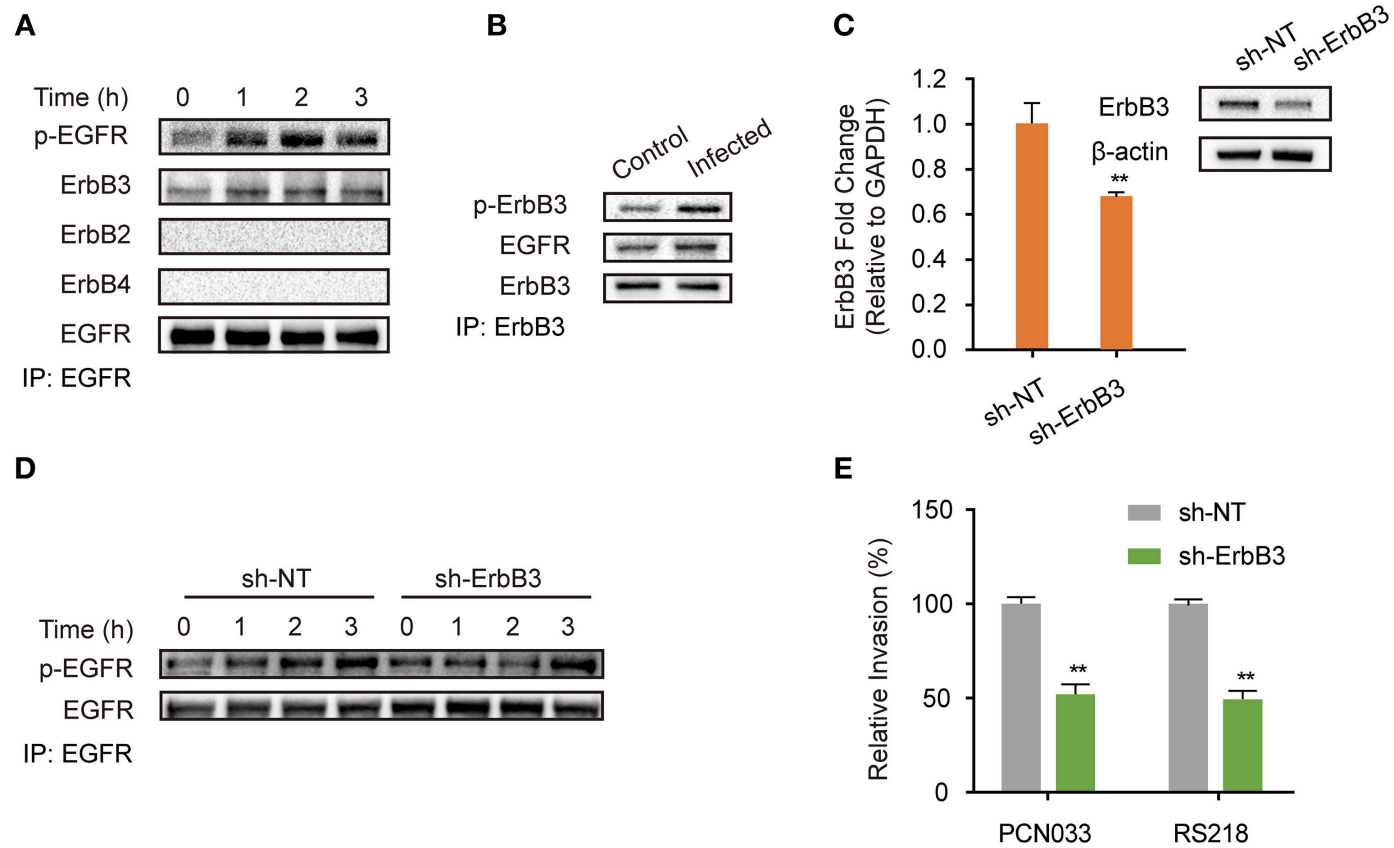
EGFR-ErbB3 dimerization induced by meningitic *E. coli* is important for EGFR activation and bacterial invasion of hBMECs. **(A)** Identification of the possible dimers involved in the EGFR response to meningitic *E. coli* infection. Infected cell lysates were immunoprecipitated with the anti-EGFR antibody, and then subjected to western blotting with different antibodies against ErbB. **(B)** Verification of the association between ErbB3 and EGFR *via* immunoprecipitation experiments with the anti-ErbB3 antibody. The hBMECs were challenged with PCN033 for 3 h. **(C)** Knock-down and verifying ErbB3 expression levels in hBMECs *via* the shRNA approach. β-actin and GAPDH were used as the endogenous controls for western blotting and quantitative PCR. **(D)** Comparison of the infection-induced EGFR activation in the control cells and the ErbB3 knocked-down cells. **(E)** Effect of the ErbB3 knock-down on meningitic *E. coli* (PCN033 and RS218 strains) invasion of hBMECs. ^**^p < 0.01.

EGFR belongs to the ErbB family of receptor tyrosine kinases, which consists of four closely related members (ErbB1/EGFR, ErbB2, ErbB3, ErbB4), and when its activation occurs it is accompanied by homodimer or heterodimer formation (Graus-Porta et al., 1997; Olayioye et al., 2000; Schlessinger, 2002; Higashiyama et al., 2008; Hofman et al., 2010; Harskamp et al., 2016; Yang X. P. et al., 2016). Therefore, we next investigated the possible dimerization of EGFR in hBMECs in response to meningitic *E. coli* infection. As shown in Figure 2A, we observed a corresponding increase of ErbB3 in the EGFR immunoprecipitated samples, accompanied with EGFR activation. In contrast, we did not find any association of ErbB2 or ErbB4 with EGFR during the infection (Figure 2A), although ErbB2 is widely reported to be a heterodimerization partner for EGFR (Graus-Porta et al., 1997), suggesting that ErbB3 might be recruited to EGFR during its phosphorylation. We also obtained a similar result with the immunoprecipitation experiments using an anti-ErbB3 antibody, which showed that the increased EGFR association level was accompanied by the phosphorylation of ErbB3 (Figure 2B). These data further support our hypothesis on the formation of EGFR–ErbB3 heterodimers in response to meningitic *E. coli* infection. ErbB3 was subsequently knocked-down in the hBMECs *via* transfection with specific short hairpin RNA (shRNA) (Figure 2C). We also observed that the time-dependent phosphorylation of EGFR in response to meningitic *E. coli* was attenuated and delayed in the sh-ErbB3 transfected cells (Figure 2D), and that invasion by the meningitic *E. coli* K1 strain (RS218) or the K2 strain (PCN033) decreased significantly upon ErbB3 knock-down (Figure 2E) compared with that in the control cells. Collectively, these results provide evidence that meningitic *E. coli* infection induced the heterodimerization of EGFR–ErbB3 in hBMECs, which is important for EGFR activation and bacterial invasion.

### EGFR Activation Is Necessary for Meningitic *E. coli*-Induced Cytoskeleton Fiber Alteration

**Figure 3:**
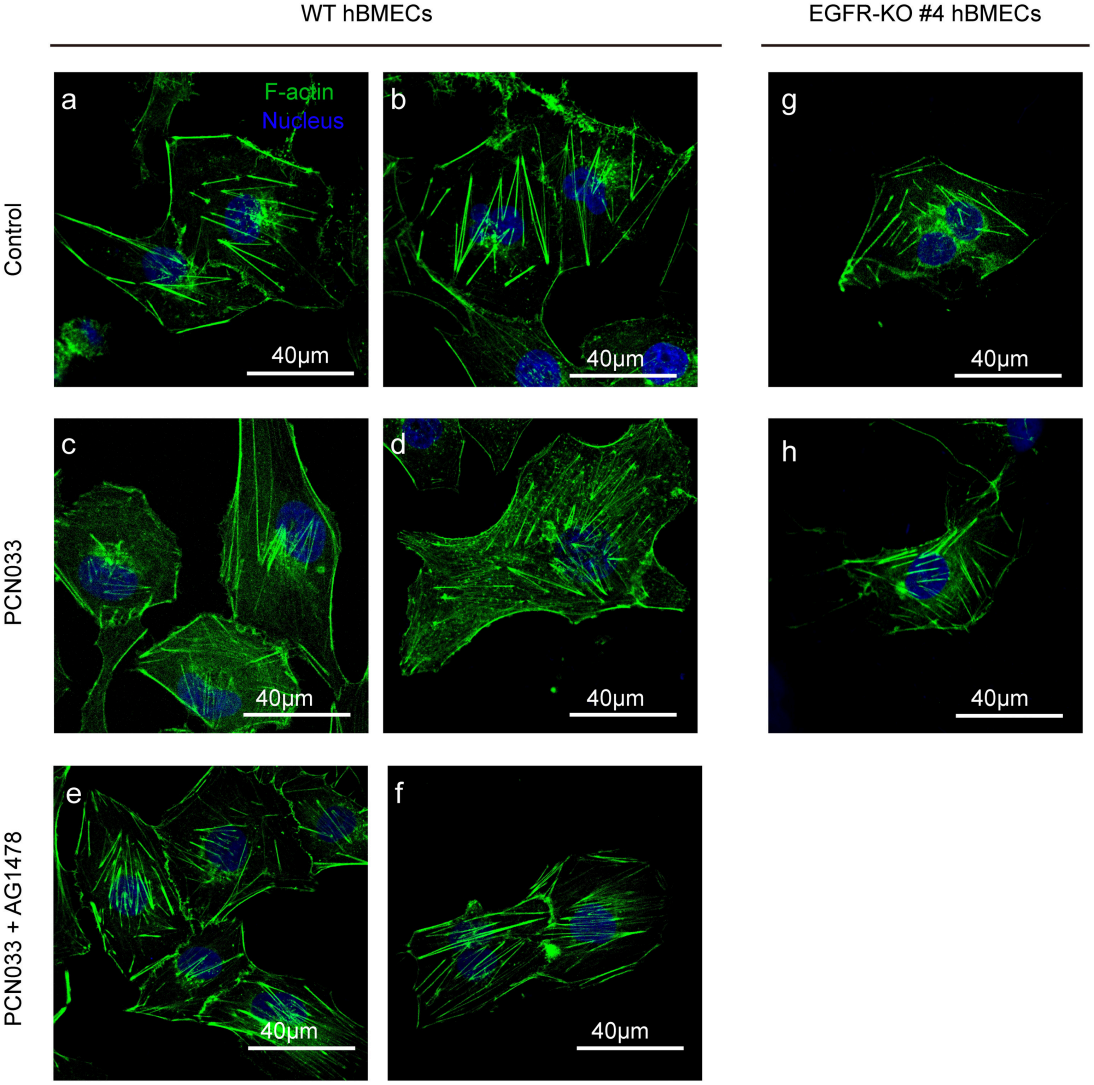
EGFR activation assists with PCN033-induced cytoskeleton fiber breakdown. The wild-type hBMECs **(a–f)** and the EGFR-KO cells (clones #4, **g,h**) were challenged with PCN033 with or without AG1478 (as described in Materials and Methods), and then subjected to confocal fluorescence microscopy. F-actin is labeled with actin-tracker green, and nuclei are stained with DAPI. The **(a–f)** were biological duplicates in each group, respectively.

We have described above the involvement of EGFR as well as its activation in meningitic *E. coli* invasion of hBMECs. Because actin cytoskeleton rearrangement is known to be one of the indispensable prerequisites for bacterial invasion (Kim, 2003), we therefore explored the possible relationship between EGFR activity and cytoskeleton alteration during meningitic *E. coli* infection. Here, in the wild-type cells without infection, the F-actin fibers were well assembled and shown to be stretched inside the cells, evenly and integrally (Figures 3a,b). Upon infection, F-actin turned to be dispersed instead of assembling as fibers and distributed fragmentarily in cytoplasm. The normal cytoskeleton was disrupted, broken-down, and the cell shape became soft (Figures 3c,d). However, when treated with EGFR kinase inhibitor AG1478, the cytoskeleton rearrangement induced by infection was almost restored, and the F-actin presented as fibers that stretched out again inside the cytoplasm (Figures 3e,f). Moreover, when EGFR was knock-out from the cells, there was no significant difference in the appearance of the cytoskeleton F-actin fibers between cells with and without PCN033 challenge (Figures 3g,h), suggesting that meningitic *E. coli*-induced alteration of cytoskeleton in hBMECs primarily requires the participation of EGFR.

### ACTN4 Acts as an EGFR Interacting Protein in hBMECs in Response to Meningitic *E. coli* Infection

**Figure 4:**
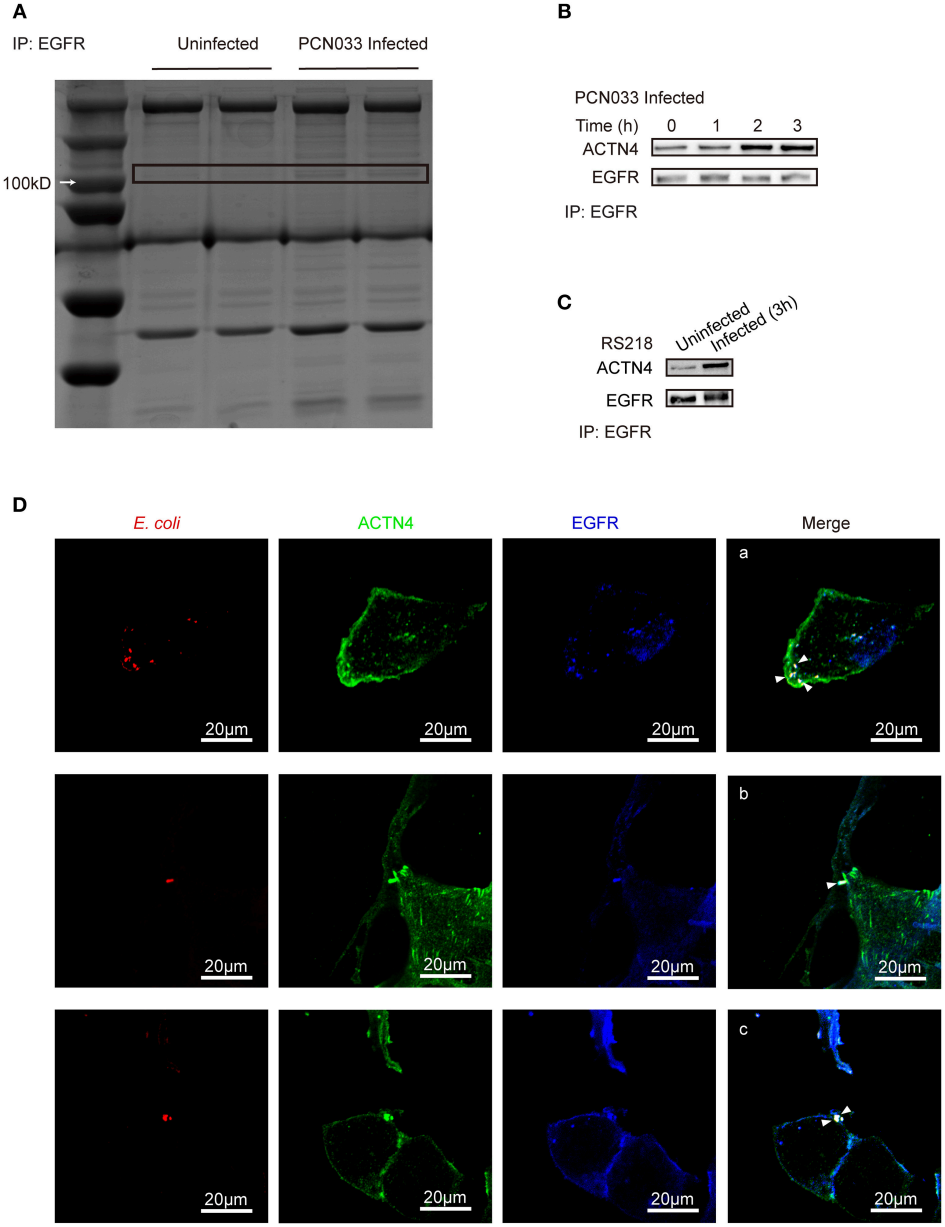
Identification of ACTN4 as an EGFR interacting protein in hBMECs upon meningitic *E. coli* infection. **(A)** SDS-PAGE comparison of the immunoprecipitation products from uninfected and infected cells. Immunoprecipitation was conducted with an anti-EGFR antibody. **(B,C)** Immunoprecipitation and western blotting verification of the EGFR–ACTN4 interaction in response to infection with meningitic *E. coli* PCN033 **(B)** and RS218 **(C)**. **(D)** Confocal immunofluorescence microscopy showed the meningitic *E. coli* co-located with EGFR and ACTN4. *E. coli* was labeled with Cy3, ACTN4 was labeled with FITC, and EGFR was labeled with Dylight 405. Cells were mounted and visualized using the Zeiss LSM 880 confocal system. The scale indicates 20 μm.

As EGFR was shown to influence the host's actin cytoskeleton, we next tried to identify the mechanism behind this phenotype. Immunoprecipitation was performed with cell lysates from both the control cells and the PCN033-infected cells with anti-EGFR antibody, and the bands obtained from sodium dodecyl sulfate polyacrylamide gel electrophoresis (SDS-PAGE) were compared (Figure 4A). Several proteins (bands) were shown to be induced by PCN033 infection, and one at around 100 kDa was identified as ACTN4 (a type of cytoskeleton-associated protein), through mass spectrum (MS) analysis (Supplementary Figure 4A), suggesting that EGFR might recruit additional ACTN4 protein upon PCN033 challenge. Subsequently, co-immunoprecipitation (Co-IP) experiments were applied to PCN033-infected cell lysates using anti-EGFR antibody, and increased binding of ACTN4 to EGFR was observed during the PCN033 infection (Figure 4B). We also investigated this interaction in hBMECs upon infection with meningitic *E. coli* K1 strain RS218, and a similar result of increased binding of ACTN4 to EGFR was also observed (Figure 4C). Interestingly, our MS data also identified the actin from EGFR immunoprecipitates (Supplementary Figure 5A), which aroused our concern that if the EGFR recruitment of ACTN4 depended on the actin. However, our western blotting result showed no significant alteration of the actin from EGFR immunoprecipitates in response to the infection (Supplementary Figure 5C, left panel), raising the speculation that this actin protein would be naturally conjugated with EGFR, and this binding might not be attributed to the recruited ACTN4. To test this hypothesis, we performed another EGFR immunoprecipitation with or without pretreatment of Cytochalasin D (CyD), a widely used actin polymerization inhibitor. The result showed that CyD treatment largely increased the EGFR recruitment of ACTN4 during the infection (Supplementary Figure 5D), which suggested that the infection-activated EGFR recruitment of ACTN4 was a direct interaction and independent of actin. Additionally, we tested the position relationship between bacteria, EGFR and ACTN4 through confocal immunofluorescence. The results showed that *E. coli* (Cy3 labeled in red), ACTN4 (FITC labeled in green) and EGFR (Dylight 405 labeled in blue) could co-locate at the edge of hBMECs (Figure 4D, bright spots with white triangle), which was highly consistent with our previous finding that EGFR could be recruited to the invasion site of meningitic *E. coli* strain (Wang et al., 2016). Therefore, these findings support the notion that ACTN4 interacts with EGFR in hBMECs in response to meningitic *E. coli* infection which is independent of the actin, and meningitic *E. coli* can facilitate this interaction during its infection of host cells.

### Transactivated EGFR Competitively Recruits ACTN4 From F-actin to Facilitate Bacterial Invasion

**Figure 5:**
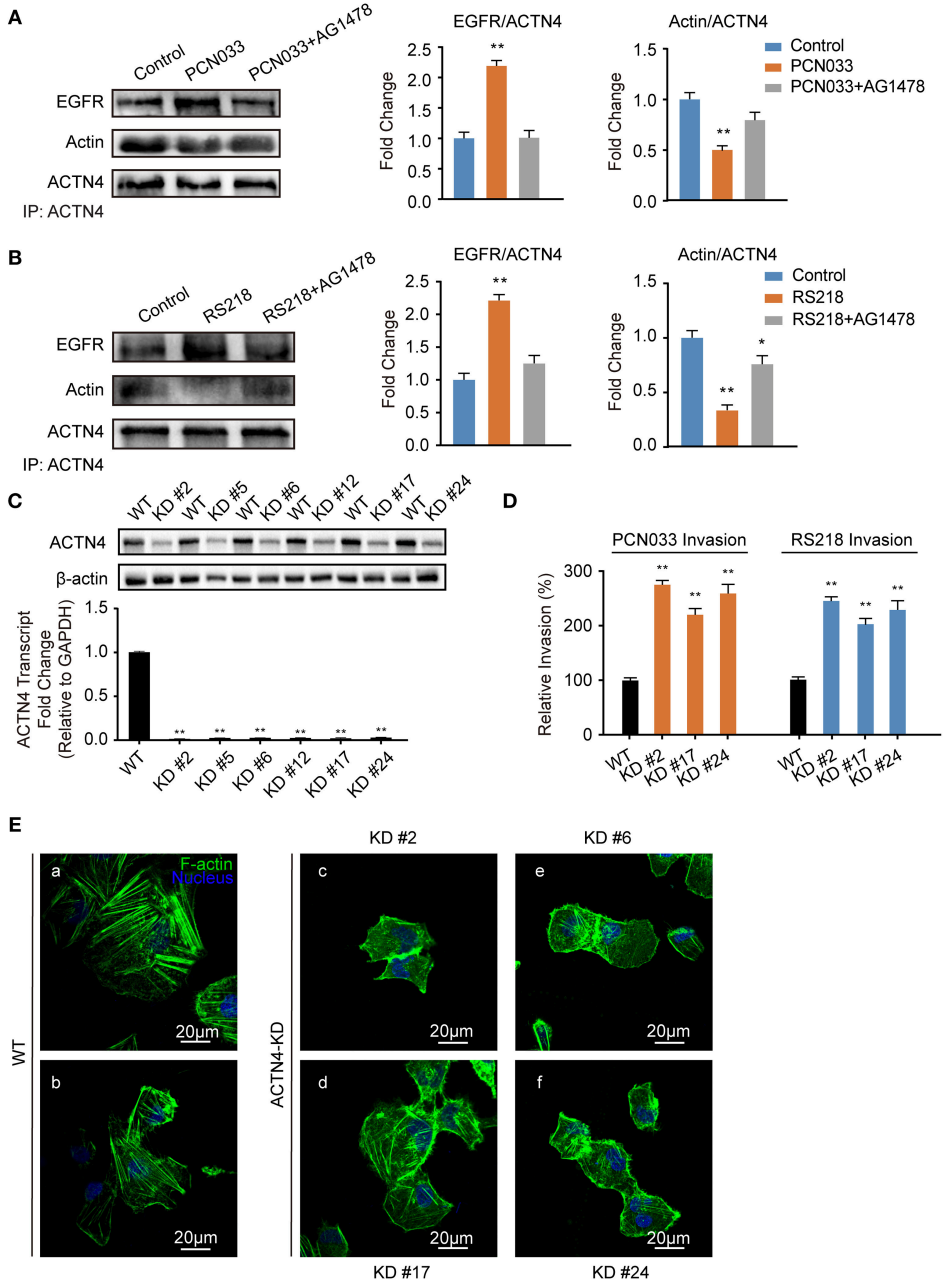
Meningitic *E. coli*-induced EGFR recruitment of ACTN4 is important for bacterial invasion. **(A,B)** The meningitic *E. coli* PCN033- **(A)** or RS218-induced **(B)** activation of EGFR results in increased recruitment of ACTN4 to EGFR and decreased binding of ACTN4 to F-actin, while inhibition of EGFR by AG1478 reversed this alteration. The densitometry results represent the ratio of EGFR to ACTN4 as well as F-actin to ACTN4. **(C)** Quantitative PCR and western blotting verification after ACTN4 genetic manipulation *via* CRISPR/Cas9. **(D)** Effect of the ACTN4 knock-down on meningitic *E. coli* PCN033 or RS218 invasion. Results are presented as the relative invasion percentage compared with that of the wild-type cells. **(E)** Effect of the ACTN4 knock-down on cell cytoskeleton morphology *via* confocal fluorescence microscopy. ^*^p < 0.05, ^**^p < 0.01.

ACTNs are a family of proteins that predominate in cross-linking actin filaments to stabilize transcellular stresses (Hsu and Kao, 2013). It has been reported a lack of ACTN4 binding to actin fibers might lead to cytoskeleton reorganization in HeLa cells (Sun et al., 2016). As we have demonstrated the participation of EGFR in the meningitic *E. coli*-induced cytoskeleton rearrangement, and also the association of EGFR with ACTN4 herein, we speculated that ACTN4 might be the middle bridge for EGFR communication with the cytoskeleton. Following immunoprecipitation with the anti-ACTN4 antibody, we found binding of EGFR to ACTN4 increased significantly in response to PCN033 infection, while contrastingly, the association of cellular actin molecules with ACTN4 decreased markedly (Figure 5A). After pretreating the cells with EGFR inhibitor AG1478, the infection-induced binding of ACTN4 to EGFR decreased, and its binding to intracellular actin was restored to that of the control level (Figure 5A). We also observed similar results in cells undergoing K1 strain RS218 infection, which showed the EGFR-related competitive recruitment of ACTN4 from actin in the cell cytoskeleton (Figure 5B). This phenotype was further evidenced by the subsequent confocal immunofluorescence (Figure 7). In the normal state, there was abundant ACTN4 protein (Cy3 labeled in red) co-located with the intracellular and out membrane F-actin fibers (FITC-phalloidin labeled in green), which were shown merged with orange coloration (Figure 7a, arrow heads indicated). During PCN033 infection, EGFR (Dylight 405 labeled in blue) was recruited and more ACTN4 became bound (shown as the color purple), while contrastingly, the co-location of ACTN4 and F-actin disappeared from view in the cells (Figure 7b, arrows indicated). When EGFR activation was inhibited, its co-location with ACTN4 was largely blocked, whereas ACTN4 and F-actin co-location was restored (Figure 7c, arrow heads). We additionally generated several ACTN4 knock-down (KD) cells through the CRISPR/Cas9 approach (Figure 5C), and found that both the K1 strain RS218 and K2 strain PCN033 of meningitic *E. coli* showed increased invasion of the ACTN4 knock-down cells, compared with that of the wild-type hBMECs (Figure 5D). Here, we presumed a morphological explanation for this increased invasion that the regular cytoskeleton and cell structures were unable to be well-maintained following ACTN4 knock-down, compared with the normal cells. As shown in Figure 5E, the F-actin structure was largely disrupted after ACTN4 knocking down. The FITC-labeled F-actin molecules intricately distributed in the cytoplasm instead of forming fibers (Figures 5Ec–f), similar to that observed in PCN033 infected cells (Figures 3c,d). To support this, we moreover employed a non-meningitic *E. coli* strain HB101, and detected its invasion of the wild type hBMECs as well as ACTN4-KD cells. As shown in Supplementary Figure 6, the invasion of hBMECs by HB101 was extremely lower compared with that by PCN033, and likewise, HB101 invasion of the ACTN4-KD cells was significantly higher than its invasion of the wild-type cells.

### CNS-Infecting *S. suis* Also Exploits EGFR Recruitment of ACTN4 to Disrupt Cytoskeleton

**Figure 6:**
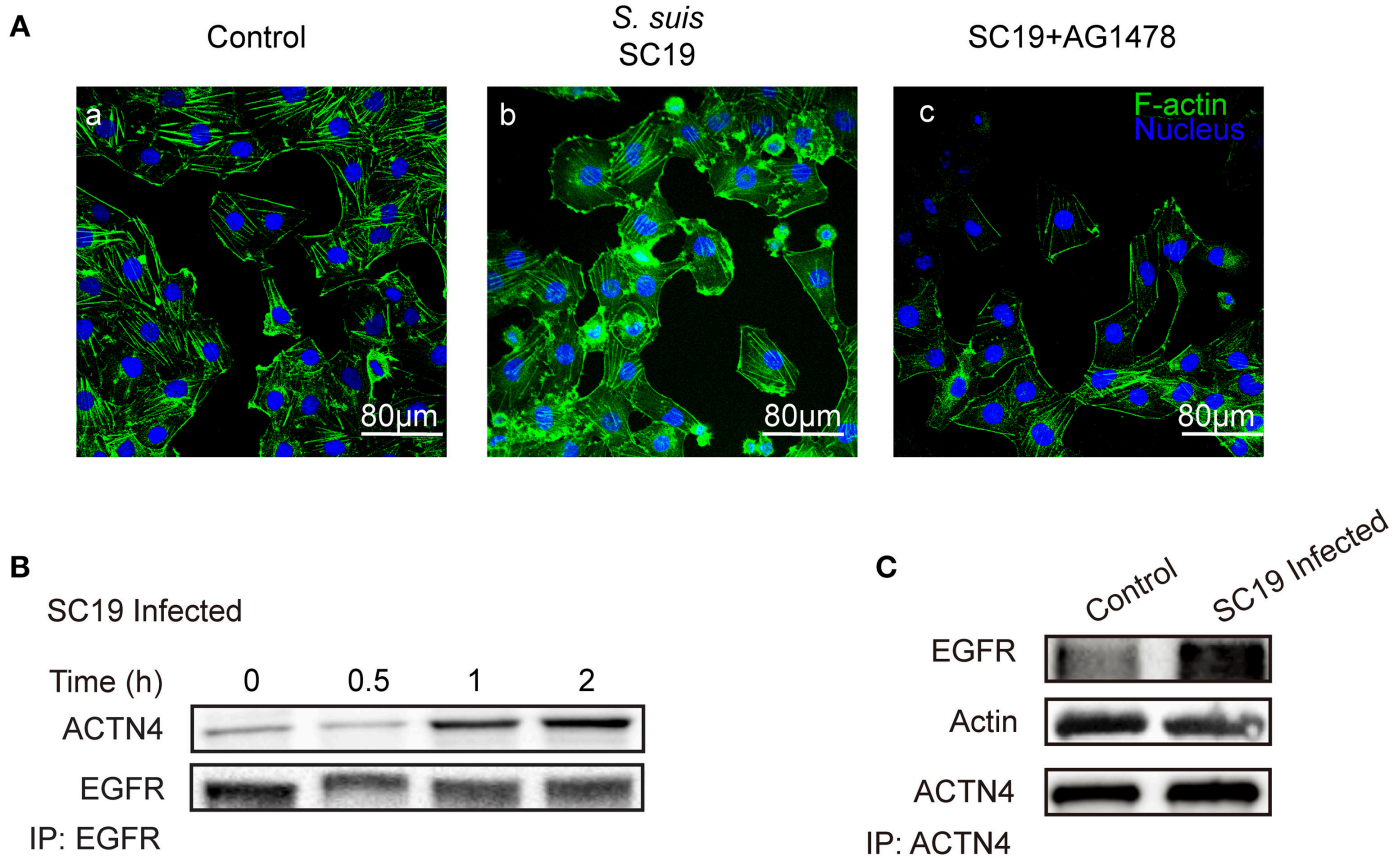
*S. suis* SC19 infection can activate EGFR and competitively recruit ACTN4. **(A)** Confocal fluorescence microscopy showing the contribution of EGFR activity to *S. suis* SC19-induced F-actin breakdown and cytoskeleton rearrangement. F-actin was labeled with actin-tracker green, and nuclei were stained with DAPI. **(B)** Immunoprecipitation and western blotting revealed the increased recruitment of ACTN4 by EGFR over time during the infection. Anti-EGFR antibody was used for the immunoprecipitation experiments. **(C)** Immunoprecipitation and western blots showing the increased EGFR association with ACTN4, and the decreased association of F-actin with ACTN4 during the challenge infection with SC19. Immunoprecipitation was carried out using an anti-ACTN4 antibody.

**Figure 7:**
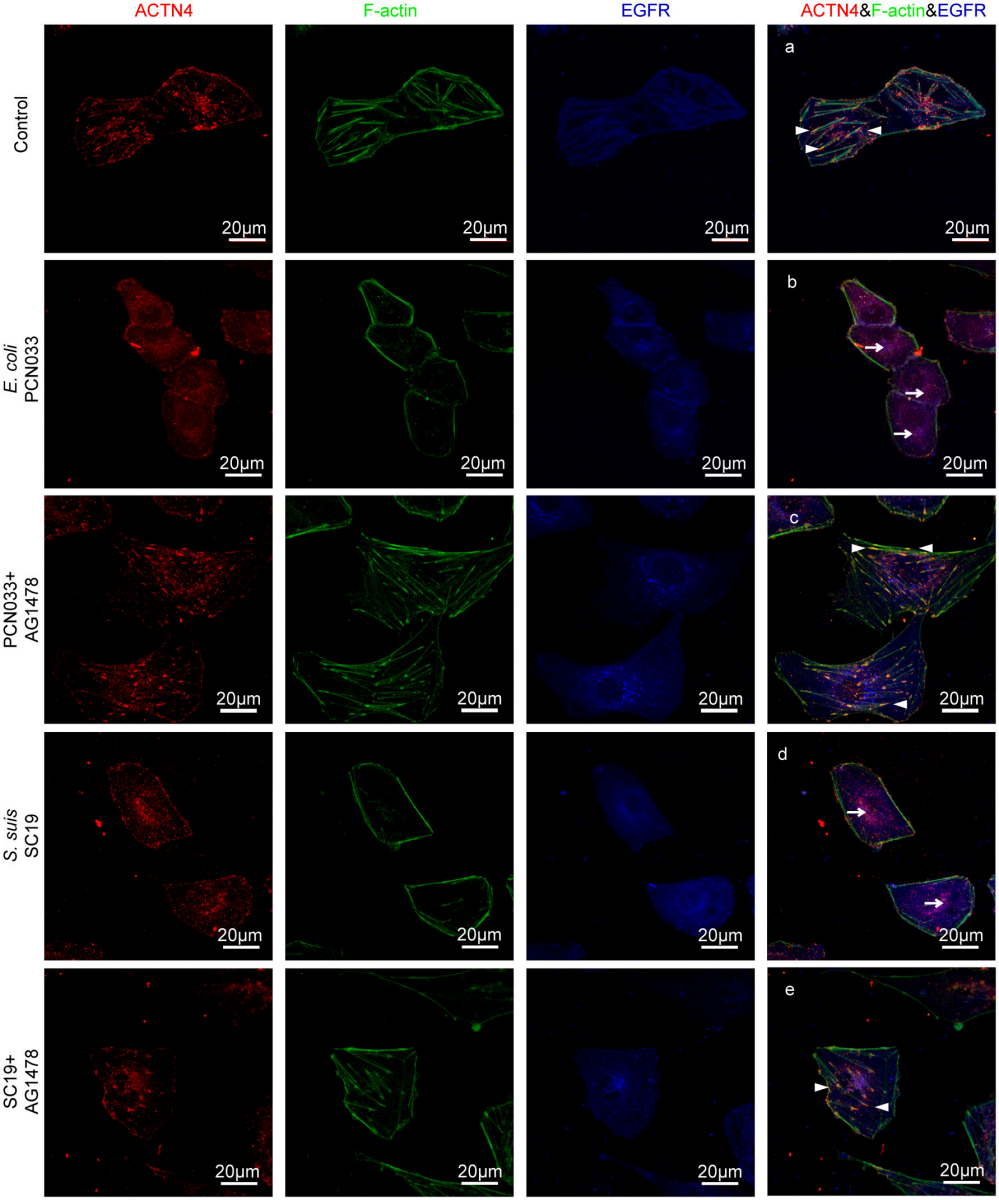
Confocal fluorescence microscopy showing activated EGFR competitively recruiting ACTN4 from intracellular F-actin in response to meningitis-causing bacteria. The meningitic *E. coli* and *S. suis* infections were conducted with or without AG1478 pretreatment of the cells as follows. **(A)** Normal hBMECs without challenge, **(B)** The cells infected with PCN033, **(C)** The cells treated with AG1478 prior to PCN033 infection, **(D)** The cells infected with SC19, **(E)** The cells treated with AG1478 prior to SC19 infection. ACTN4 was labeled with Dylight 405, EGFR was labeled with Cy3, and F-actin was stained with actin-tracker green. The arrow heads indicate the co-location of ACTN4 and F-actin, and the arrows indicate the co-location of ACTN4 and EGFR. The cells were mounted and then visualized using the Zeiss LSM 880 confocal system.

Having shown that the same mechanism involving EGFR and ACTN4 is exploited by both meningitic *E. coli* K1 and K2 strains, we further questioned whether these proteins might be the common targets for other CNS-invading bacteria. In a previous publication we have shown the Gram-positive bacterium *S. suis*, which possesses the zoonotic potential and the ability to cause meningitis, was able to transactivate host EGFR for the development of neuroinflammatory response in BMECs (Yang X. P. et al., 2016). Herein, *via* confocal imaging, we observed that infection of hBMECs with the *S. suis* serotype 2 strain SC19 disrupted the cytoskeleton, whereby the F-actin fibers became fractured and disordered compared with those of the control cells. However, when EGFR activity was inhibited by AG1478, the SC19-induced F-actin damage appeared to be largely blocked (Figure 6A). We similarly identified one EGFR interacting protein as ACTN4 through our MS analysis (Supplementary Figure 4B), and the Co-IP assay we performed with the anti-EGFR antibody showed a substantial increase in the recruitment of ACTN4 to EGFR (Figure 6B). Likewise, *via* immunoprecipitation with the anti-ACTN4 antibody, we observed an increased association of EGFR with ACTN4, and decreased binding of cellular actin to ACTN4 (Figure 6C), and this competitively recruitment was also independent of the actin (Supplementary Figures 5B,C). These findings were further evidenced by the confocal immunofluorescence microscopy, which showed a higher degree of EGFR–ACTN4 co-location in response to *S. suis* SC19 infection (Figure 7d, purple indicated by arrows), and a higher degree of ACTN4–F-actin co-location in cells following AG1478 treatment, a finding similar to the situation in control cells (Figures 7a,e, orange indicated by arrow heads). These findings support the proposition that EGFR recruitment of ACTN4 may also be utilized by meningitic *S. suis* for infection-induced cytoskeleton disorder and damage.

## Discussion

Successful penetration of the BBB by meningitis-causing bacterial pathogens requires four prerequisites: a high level of bacteremia, bacterial adhesion and invasion of BMECs, actin cytoskeleton rearrangement, and bacterial vitality maintenance (Kim, 2002, 2008; Kim et al., 2005; Coureuil et al., 2017); all of which are complex bacteria-host interactions involving multiple host molecules. EGFR is a host factor that has been found to play essential roles in several bacterial infections recently, although it was widely reported previously to be associated with different cancers (Mikami et al., 2005; Yan et al., 2009; Adams et al., 2017; Araki et al., 2017; Chandrasekaran et al., 2017; Dheeraj et al., 2017). For instance, *H. pylori* was shown to induce EGFR transactivation in gastric epithelial cells, resulting in the upregulation of MMP10 and extracellular matrix disorder (Yan et al., 2009). EGFR activation by non-typable *H. influenzae* was found to attenuate the host's immune defenses by negatively regulating the expression of Toll-like receptor 2 (Mikami et al., 2005). In our previous work, we found that invasion of hBMECs by meningitic *E. coli* K1 RS218 triggered the activation of EGFR through SphK2-S1P-S1P_2_ signaling (Wang et al., 2016). Furthermore, we found that the *S. suis* serotype 2 strain also induced EGFR activation, as well as dimerization of EGFR to mediate neuroinflammatory responses during its infection (Yang X. P. et al., 2016). From this perspective, we would like to raise the idea that host EGFR can act as the common target for multiple types of infectious bacteria during the invasion process.

It was shown previously that *E. coli* strains with K1 capsules were able to invade and survive in BMECs during penetration of the BBB (Huang et al., 1995, 1999; Kim et al., 2005). However interestingly, our recent work provided various evidences that some non K1-capsular strains, such as the PCN033 strain used herein, which is an *E. coli* K2-capsular strain isolated from diseased pig brain (Liu et al., 2015), also possess the ability to invade and penetrate the BBB *in vitro* and *in vivo* (Yang et al., 2016b). We actually compared the pathogenicity of both K1 strain RS218 and K2 strain PCN033 in our early work, and found that both strains were highly virulent and possess the ability to invade the brain and cause neurological symptoms (Yang et al., 2016b). We moreover performed the comparative genomics analysis on both strains and identified certain common features such as the presence of the Type VI secretion system, etc. (Liu et al., 2015; Peng et al., 2016). In the present study, we also observed that the K2 strain PCN033 hijacked EGFR for its invasion of hBMECs, as demonstrated by the fact that PCN033 could transactivate EGFR in a time-dependent manner, and inhibition of EGFR tyrosine activity as well as CRISPR knock-out of EGFR significantly decreased the level of bacterial invasion. Additionally, the PCN033-induced EGFR activation required the cleavage and release of its specific ligand, HB-EGF, which has also been evidenced in our early work with the *E. coli* K1 strain (Wang et al., 2016). These observations prompt us to say that *E. coli* K1- and K2-capsular strains should share certain mechanisms involving EGFR for their invasion of BMECs.

EGFR belongs to the four-member family of receptor tyrosine kinases consisting of EGFR (ErbB1), ErbB2, ErbB3 and ErbB4, which share similar molecular structures with an ectodomain providing the ligand-binding site, a single transmembrane domain, and a large cytoplasmic region containing multiple tyrosine phosphorylation sites that determine their kinase activities (Yarden and Sliwkowski, 2001; Citri and Yarden, 2006; Linggi and Carpenter, 2006). Receptor activation is initiated by dimerization events (Hofman et al., 2010), which include both homodimerization (e.g., ErbB1/ErbB1) and heterodimerization, particularly for ErbB1, ErbB3, and ErbB4 with ErbB2 (Olayioye et al., 2000; Schlessinger, 2002). We therefore investigated the possible dimerization forms in response to meningitic *E. coli* infection, and an obvious heterodimerization of EGFR and ErbB3 was observed in the hBMECs upon infection. Noticeably in Figure 2D, EGFR phosphorylation occurred in the sh-ErbB3 cells at the final time point (3 h) compared with that in the control cells. At this time point, it is hard to tell whether the delayed EGFR phosphorylation we observed results from insufficient knock-down of ErbB3, or whether some degree of EGFR homodimerization existed in the hBMECs in response to meningitic *E. coli* infection. Regardless, the EGFR–ErbB3 heterodimerization we observed was essential for the infection-induced EGFR activation as well as for bacterial invasion of the hBMECs because the ErbB3 knock-down significantly delayed and attenuated these phenotypes.

Dimerization and transactivation of EGFR require interactions to occur with its specific ligands (Schlessinger, 2002; Liebmann, 2011; Wang, 2016), one of which has been identified herein as HB-EGF in hBMECs with meningitic *E. coli* PCN033 infection. EGFR transactivation occurs by proteolytic processing of the ligand precursor, which requires the participation of ADAM activity, the so-called “Triple Membrane Passing Signal” mechanism (Wang, 2016). That is, ADAMs cleave their ligand precursors and allow the shed ligands to bind to the extracellular ligand-binding domain of EGFR, thus transactivating EGFR signaling (Jones et al., 2016). Therefore, the ADAM-mediated shedding of EGF-like ligands might serve as a key step in EGFR transactivation. Through real-time PCR, we have shown that ADAM17 was the key player in mediating EGFR activation, by the demonstration that ADAM17 inhibitor TAPI-1 treatment significantly suppressed the infection-induced transactivation of EGFR by preventing the proteolysis and secretion of HB-EGF. Taken together, these findings support the notion that meningitic *E. coli* infection induces the upregulation of and subsequent ADAM17-mediated proteolysis and secretion of HB-EGF, which results in the ligand-dependent EGFR transactivation as well as heterodimerization of EGFR–ErbB3, thus facilitating bacterial invasion of hBMECs.

Next, the question rises of how EGFR works to contribute to bacterial invasion. Previous studies have largely reported that meningitic *E. coli* can trigger the activation of multiple signaling transduction pathways for successful cell invasion (Kim, 2008), and signaling molecules such as FAK, phosphatidylinositol-3-kinase, Rho GTPases, and cytosolic phospholipase A2, among others, have all been identified as being involved in bacterial penetration of the BBB, most likely through their promotion of actin cytoskeleton rearrangement in BMECs (Reddy et al., 2000; Khan et al., 2002, 2003, 2007; Shin et al., 2005; Zhu et al., 2010a,b). Here, by comparing the anti-EGFR immunoprecipitation products from cells with or without infection, we identified ACTN4 as an interacting protein for EGFR. ACTN4 is known to cross-link intracellular actin filaments to maintain the stable cellular morphology of the cytoskeleton (Yao et al., 2011). More interestingly, we observed that activated EGFR could act as a sponge to recruit ACTN4 from F-actin, and knock-down of ACTN4 in wild-type cells significantly increased F-actin fibers disruption and cytoskeleton alteration. We also observed that meningitic *E. coli* invasion of the ACTN4 knocked-down cells was significantly higher than that of the wild-type cells, which is highly consistent with the concept that cytoskeleton rearrangement is critical for the bacterial invasion process (Kim, 2002; Colonne et al., 2016; Kühn and Mannherz, 2017). Noticeably, previous publications have widely shown that meningitic *E. coli* infection could lead to the formation of the stress fibers, ruffle, or lamellipodia, which were resulted from the actin recruitment as well as cytoskeleton rearrangement (Khan et al., 2002; Zhao et al., 2010; Loh et al., 2017). But in current study, we demonstrated that meningitic *E. coli* infection caused an increased recruitment of ACTN4 to the activated EGFR which is independent of the actin, and the actin fibers might become disrupted or fragmental without this “scaffold protein” ACTN4, which is consistent with the finding in another study (Sun et al., 2016). At this point, we have no idea whether the disrupt actin participated in these stress fibers formation or not because we mainly focused on the global intracellular alterations while those previous work mostly paid attention to the particular morphological changes on the edge of a cell. Whatever, these new findings reveal the precise downstream molecular processes that promote invasion of meningitic *E. coli* strains following EGFR activation, which entails recruitment of ACTN4 from cellular F-actin fibers and results in the breakdown as well as the rearrangement of the cytoskeleton, thus contributing to bacterial invasion. The concept was further supported by the invasion result of a negative-invasion strain HB101, which exhibited significant increased invasion once the ACTN4 was knocked down (Supplementary Figure 6). Additionally, as we have found that EGFR activation by the meningitis-causing *S. suis* type 2 strain also occurs (Yang X. P. et al., 2016), we have increasingly wondered whether this mechanism also exists during *S. suis* invasion of BMECs. Similar to what we observed in *E. coli* PCN033 and HB101, the invasion of *S. suis* strain SC19 into the ACTN4-KD cells was also much higher than its invasion of the wild-type cells (Supplementary Figure 6). By using confocal microscopy, we also found the cytoskeleton rearrangement in hBMECs during *S. suis* infection, and similarly, we observed that the infection-activated cellular EGFR competitively recruited ACTN4 from F-actin, which accounts for the infection-induced cytoskeleton disturbance. However, the detailed connection between EGFR activation and ACTN4 recruitment still remains incompletely understood and needs further investigation.

**Figure 8:**
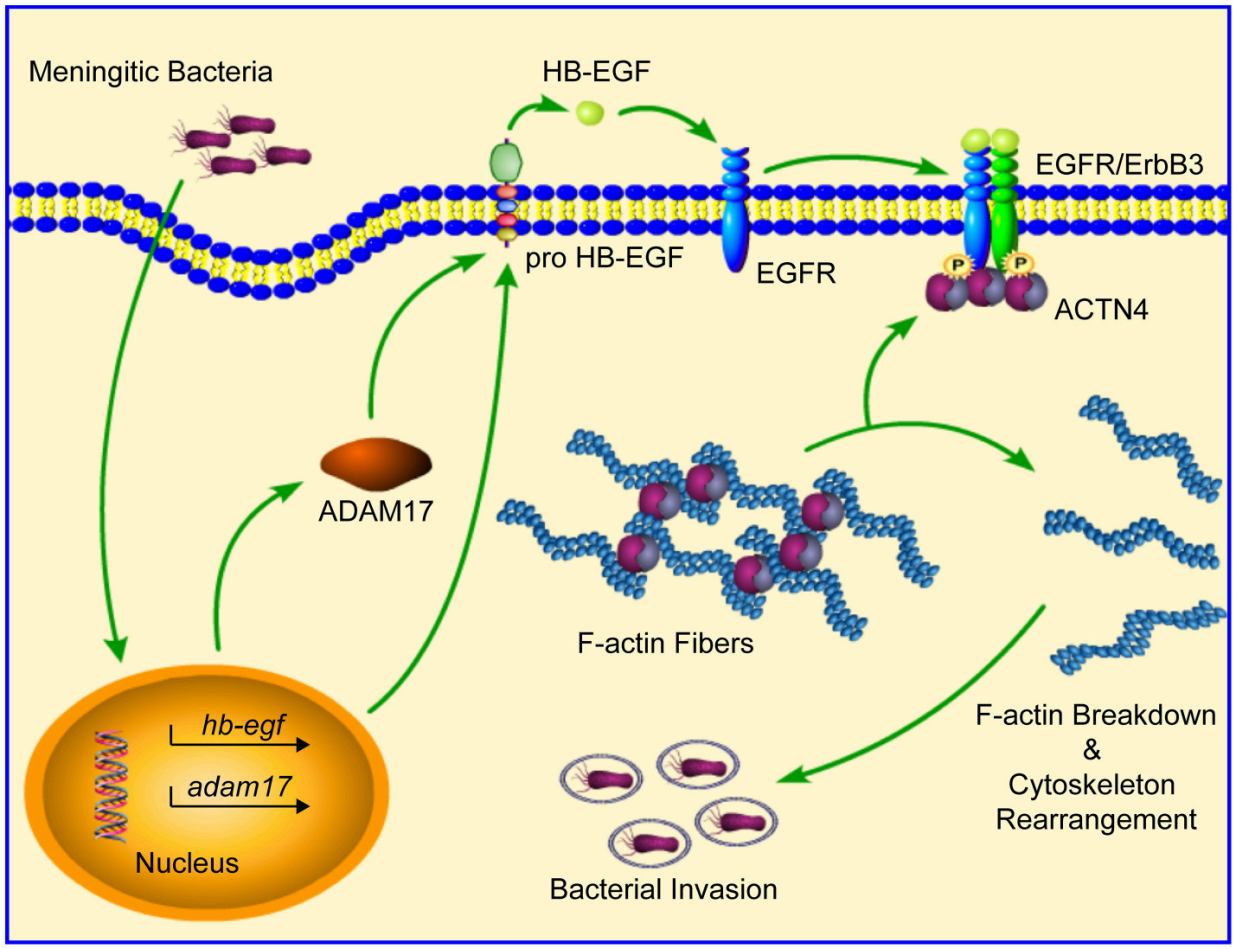
Schematic representation of the mechanism involving EGFR in meningitic bacterial penetration of the BBB. Bacterial infection of hBMECs induces the ligand (HB-EGF)-dependent transactivation of EGFR, which requires the cleavage activity of ADAM17. Activated EGFR dimerizes with its heterogenous partner ErbB3, and then competitively recruits and disassembles ACTN4 from the intracellular F-actin fibers, leading to breakdown as well as reorganization of the actin cytoskeleton, which eventually facilitates bacterial invasion.

In summary, our current work provides further support for the essential role played by EGFR in meningitic *E. coli* penetration of the BBB by elucidating its mechanism of action. Specifically, our study has shown that bacterial infection of hBMECs is able to induce a ligand-dependent transactivation of EGFR, which requires the ADAM17-mediated cleavage and release of HB-EGF. The transactivated EGFR subsequently competitively recruits and disassembles ACTN4 from intracellular F-actin fibers, resulting in the rearrangement of the actin cytoskeleton, thereby eventually facilitating bacterial invasion (Figure 8). Notably, this strategy involving activated EGFR competing with F-actin for ACTN4 to disrupt the cytoskeleton is exploited not only by meningitic *E. coli* K1 and K2 strains, but also by meningitis-causing *S. suis* strains. To the best of our knowledge, this is the first demonstration that CNS-infecting pathogens exploit EGFR-ACTN4 for their successful invasion of the BBB, suggesting that the EGFR-ACTN4 cascade may offer targets for the better prevention and treatment of bacterial meningitis.

## Author Contributions

JF and XW conceived and designed the research. JF, LLi, XY, RY, and NA performed the experiments. JF, XW, LLiu, and CT analyzed the data. CT, HC, and XW contributed reagents, materials, analysis tools. JF and XW drafted and revised the paper.

### Conflict of Interest Statement

The authors declare that the research was conducted in the absence of any commercial or financial relationships that could be construed as a potential conflict of interest.
