# Supplementary material for: Transactivated Epidermal Growth Factor Receptor Recruitment of α-actinin-4 From F-actin Contributes to Invasion of Brain Microvascular Endothelial Cells by Meningitic Escherichia coli
Source: Front Cell Infect Microbiol. 2019 Jan 9;8:448. doi: 10.3389/fcimb.2018.00448 (PMC6333852; doi:10.3389/fcimb.2018.00448)
Supplement: Supplementary file 1 [file Data_Sheet_1.docx]

**Supplementary Figures**

**Supplementary Figure 1**

**
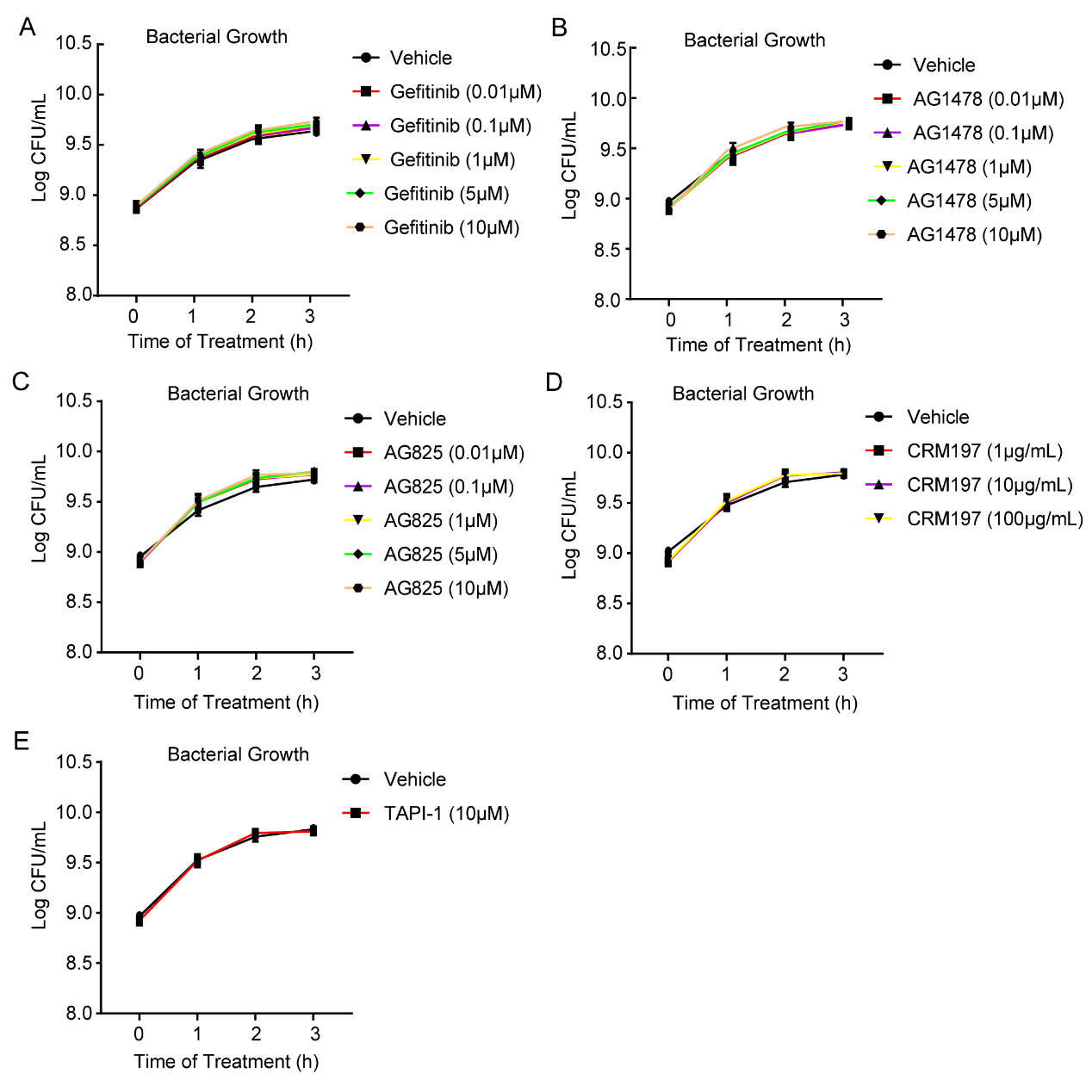
**

**Supplementary Figure 1. Bacterial growth was not affected by the treatment of chemicals.** Overnight bacterial culture was diluted and resuspended in fresh medium at the OD_600_ of 0.8. The suspension was subjected to the incubation with or without inhibitors at indicated concentrations for 3 hours (**A**, Gefinitib; **B**, AG1478; **C**, AG825; **D**, CRM197; **E**, TAPI-1). Viable bacterial counts were determined by series dilution and plating at 1 h of interval, and expressed as Log CFU/mL.

**Supplementary Figure 2**

**
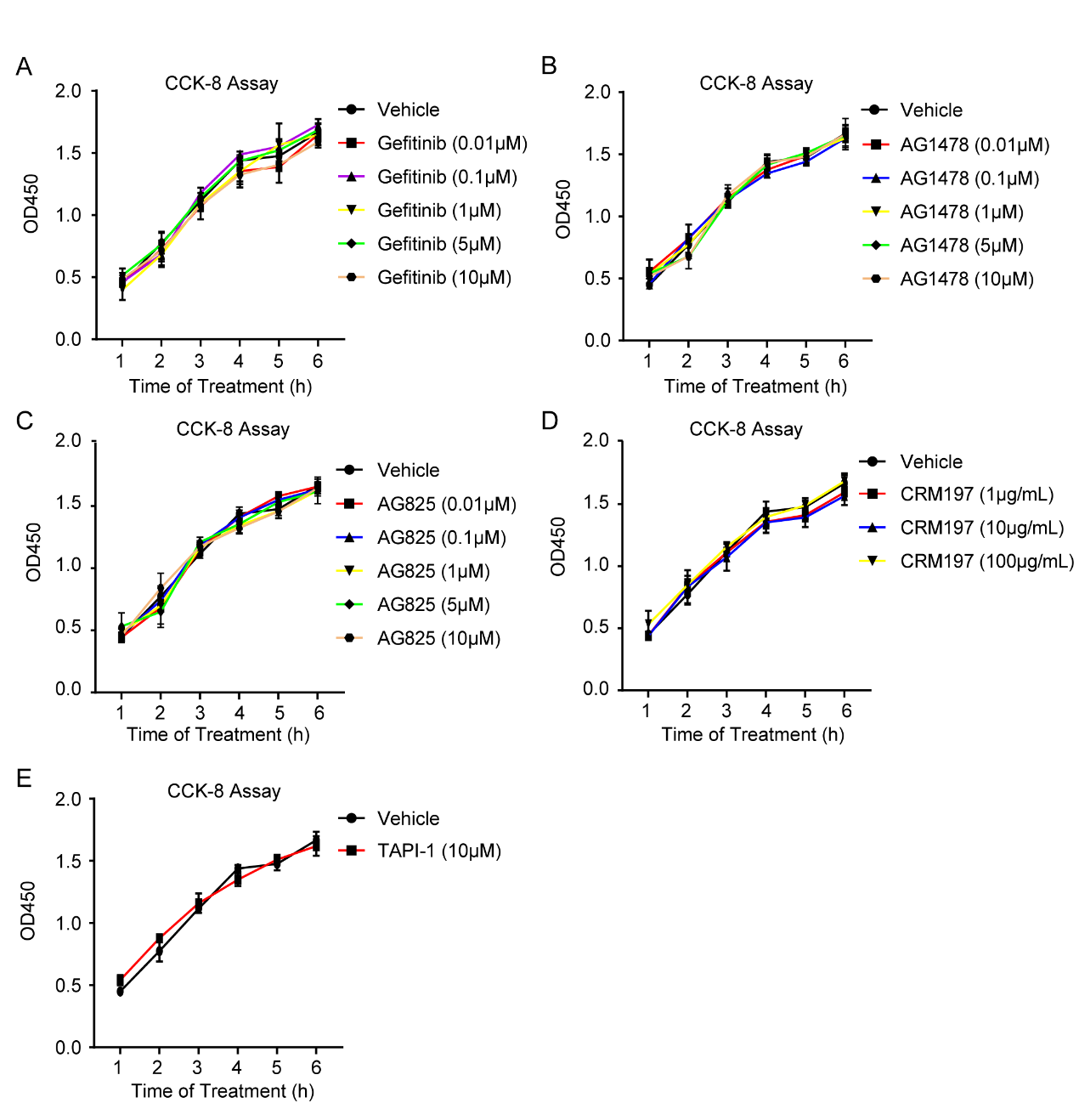
**

**Supplementary Figure 2. The chemicals used in this study did not show toxic effects on viability of the hBMECs via the CCK-8 assay.** The chemicals are gefitinib **(A)**, AG1478 **(B)**, AG825 **(C)**, CRM197 **(D)** and TAPI-1 **(E)**.

**Supplementary Figure 3**

**
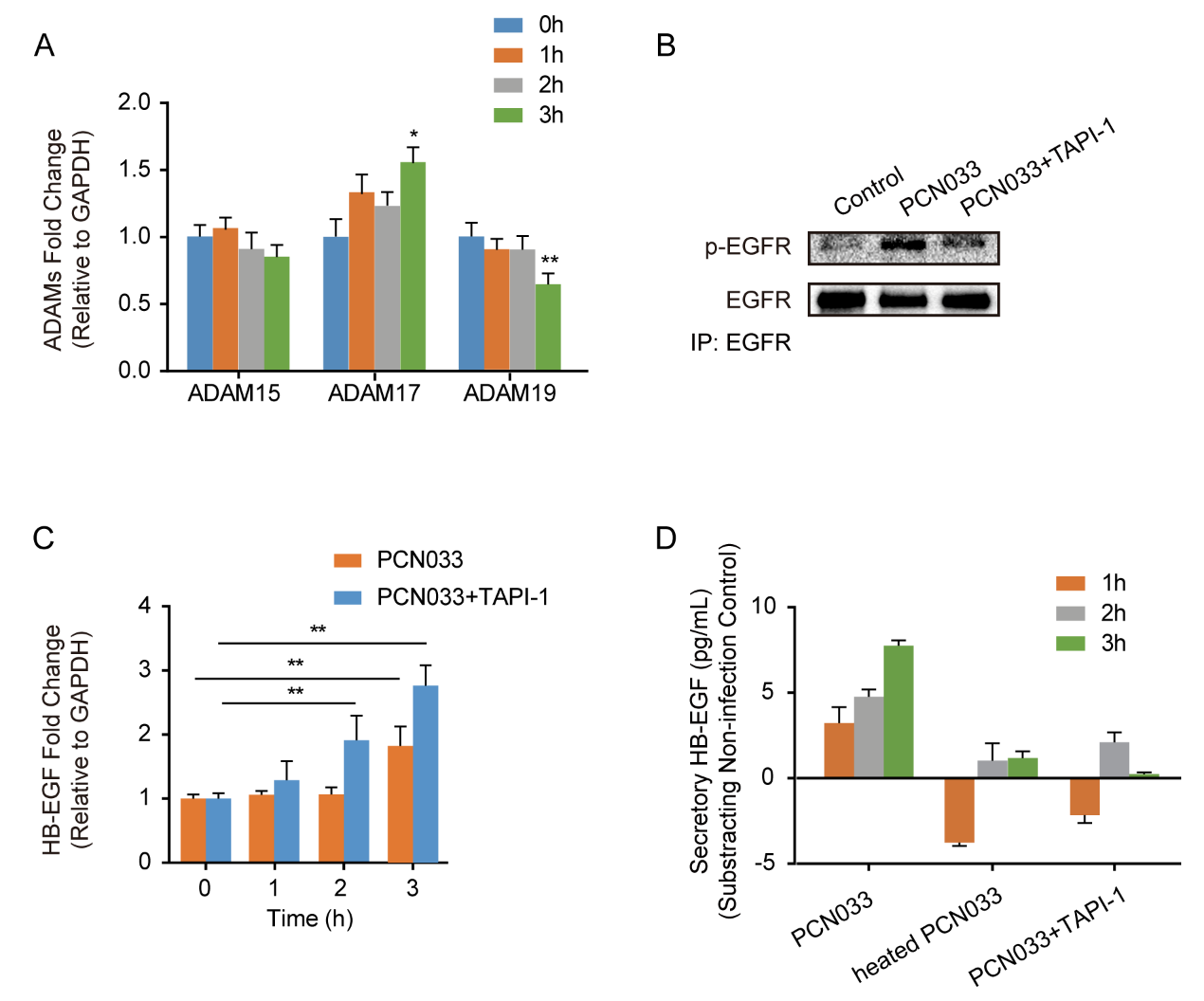
**

**Supplementary Figure 3. ADAM17 participates in meningitic *E. coli*-induced EGFR activation. (A)** Quantitative PCR identification of ADAM17 upregulation in response to meningitic *E. coli* PCN033. GAPDH was used as the endogenous control. **(B)** Effect of the ADAM17 inhibitor TAPI-1 treatment on PCN033-induced EGFR activation. **(C-D)** The effects of TAPI-1 treatment on PCN033-induced transcription (C) as well as release (D) of the HB-EGF in the hBMECs.

**Supplementary Figure 4**

**
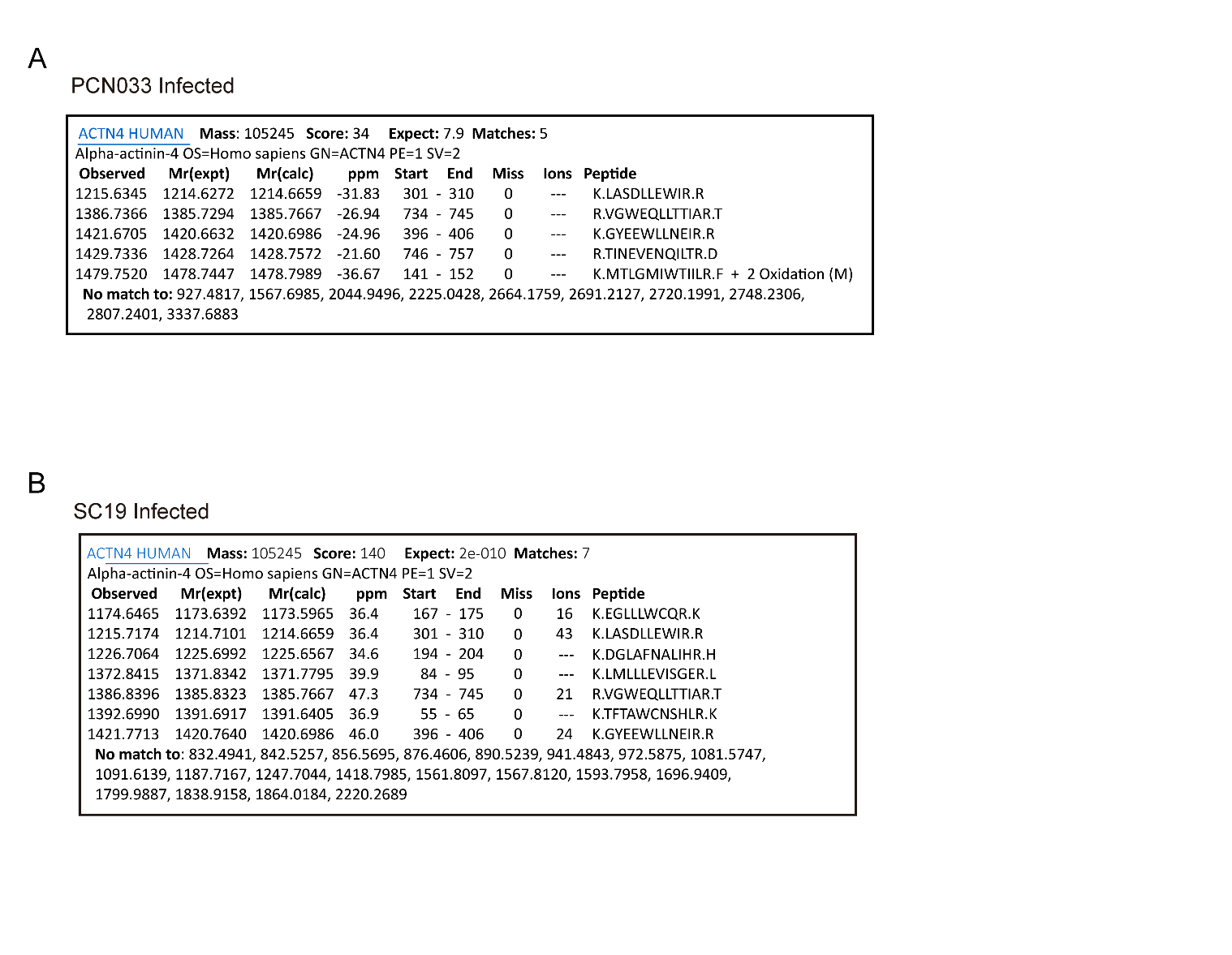
**

**Supplementary Figure 4.** ACTN4 was identified *via* MS from EGFR immunoprecipitates in hBMECs infected by meningitc *E. coli* (**A**) or *S. suis* (**B**).

**Supplementary Figure 5**

**
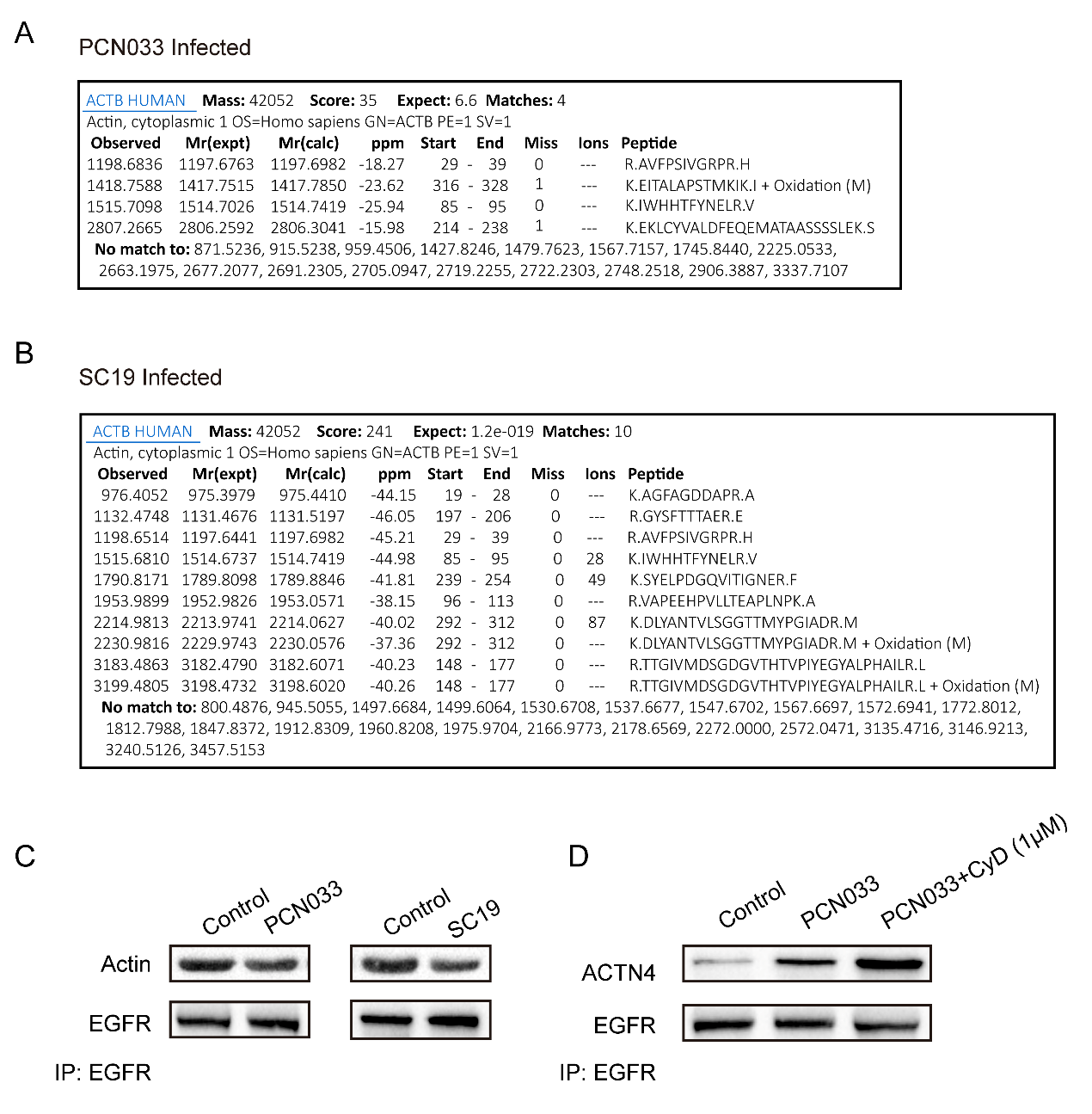
**

**Supplementary Figure 5. Infection-activated EGFR recruitment of ACTN4 was independent of actin. (A-B)** Actin was identified *via* MS approach from EGFR immunoprecipitates of the cells infected by meningitic *E. coli* PCN033 **(A)** or *S. suis* strain SC19 **(B)**. **(C)** Immunoprecipitation and Western blotting verification of the actin binding with EGFR in response to meningitic *E. coli* PCN033 and *S. suis* SC19. **(D)** The effect of CyD (1μM) treatment on the EGFR recruiting ACTN4 in response to *E. coli* infection evaluated by immunoprecipitation and Western blotting.

**Supplementary Figure 6**

**
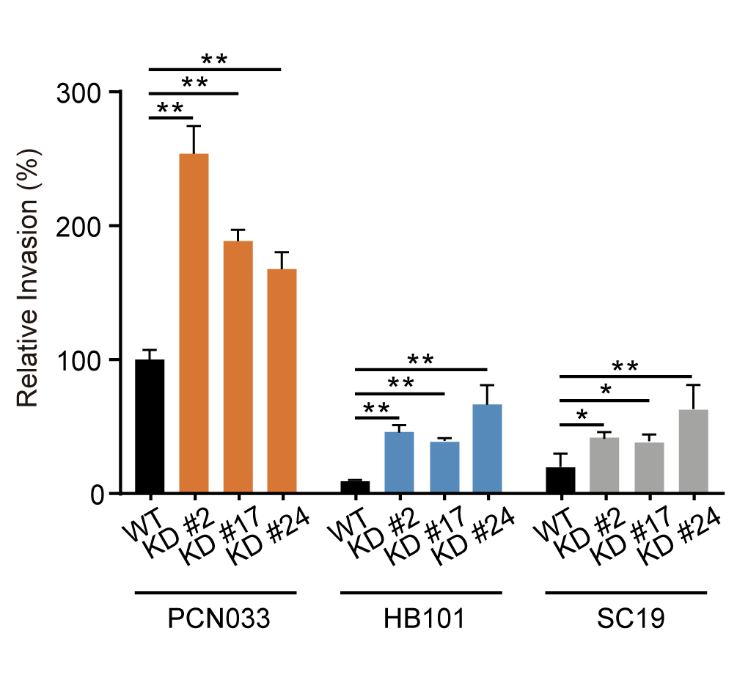
**

**Supplementary Figure 6. ACTN4 knocking-down in hBMECs significantly increased bacterial invasion.** Data were calculated as mean ± SD of three duplications and presented as relative invasion compared with the invasion of wild-type cells by PCN033. * *p*<0.05, ** *p*<0.01.

**Supplementary Table.**

**Supplementary Table 1. The list of primers used for real-time PCRs.**

| Name | Sequence (5’---3’) |
| --- | --- |
| EGFR-F | TGCCACCTGTGCCATCCA |
| EGFR-R | ACCACCAGCAGCAAGAGGAG |
| ErbB3-F | GTGGCAGAGGGCAAGTGT |
| ErbB3-R | GGTGGCAGGAGAAGCATTCG |
| AREG-F | ATTATGCTGCTGGATTGG |
| AREG-R | GAGGACGGTTCACTACTA |
| BTC-F | CCAAGCAATACAAGCATTAC |
| BTC-R | GAGGACGGTTCACTACTA |
| EREG-F | AGTTCAGACAGAAGACAATC |
| EREG-R | ACATCGGACACCAGTATA |
| HB-EGF-F | TATACCTATGACCACACAAC |
| HB-EGF-R | CACATCATAACCTCCTCTC |
| TGF-α-F | GGCTGTCCTTATCATCAC |
| TGF-α-R | AGACCACTGTTTCTGAGT |
| ADAM15-F | GTGGAGTTGGTGATTGTG |
| ADAM15-R | GAATGTGTCCAGCAAGAG |
| ADAM17-F | TCGCATTCTCAAGTCTCC |
| ADAM17-R | GCAACATCTTCACATCCC |
| ADAM19-F | TTGCCGAGGAATTAGAGG |
| ADAM19-R | GCTTCTTGGTCTGTTGTG |
| ACTN4-F | GATGGAGGACTACGAGAAG |
| ACTN4-R | CAGCGTGTTGAAGTTGAT |
| GAPDH-F | TGCCTCCTGCACCACCAACT |
| GAPDH-R | CGCCTGCTTCACCACCTTC |
|  |  |
